# Cortical thickness signature of early-onset Alzheimer’s disease across clinical stages and A/T1 biomarker profiles

**DOI:** 10.1007/s00415-026-14187-0

**Published:** 2026-10-07

**Authors:** Alessandro Zilioli, Rosaleena Mohanty, Anna Rosenberg, Anna Matton, Tobias Granberg, Göran Hagman, Marco Spallazzi, Miia Kivipelto, Eric Westman

**Affiliations:** 1 Department of Neurology, University-Hospital of Parma, Parma, Italy; 2 Division of Clinical Geriatrics, Center for Alzheimer Research, Department of Neurobiology, Care Sciences and Society, Karolinska Institutet, Huddinge, Stockholm, Sweden; 3 Department of Public Health, Finnish Institute for Health and Welfare, Helsinki, Finland; 4 Ageing Epidemiology Research Unit, School of Public Health, Imperial College London, London, UK; 5 Department of Clinical Neuroscience, Karolinska Institutet, Stockholm, Sweden; 6 Department of Neuroradiology, Karolinska University Hospital, Stockholm, Sweden; 7 Theme Inflammation and Aging, Karolinska University Hospital, Stockholm, Sweden; 8 Institute of Public Health and Clinical Nutrition, University of Eastern Finland, Kuopio, Finland

**Keywords:** Early-onset Alzheimer’s disease, Cortical thickness, A/T1 biomarker profiles, Mild cognitive impairment, Subjective cognitive decline

## Abstract

**Background:**

The cortical thinning signature of early-onset Alzheimer’s disease (EOAD) is well established in dementia, but its emergence during earlier disease stages remains unclear. We investigated when it becomes detectable and whether it differs according to fluid amyloid and phosphorylated-tau (A/T1) biomarker profiles.

**Methods:**

We studied 292 individuals <65 years who underwent 3T structural MRI and cerebrospinal fluid assessment of Aβ42/40 and *p*-tau181. Participants were classified as A−T1− subjective cognitive decline (SCD; reference group), A+T1−, or A+T1+. Cortical thickness and hippocampal volume were measured using FreeSurfer. EOAD signature regions were defined by comparing biomarker-confirmed AD dementia with the reference group and subsequently examined across A/T1 profiles in SCD and mild cognitive impairment (MCI). Brain–cognition associations were assessed.

**Results:**

The cohort comprised 107 reference and 185 amyloid-positive participants (76 A+T1− and 109 A+T1+). EOAD dementia was characterized by marked cortical thinning in posterior parietal and lateral temporal regions. In MCI, cortical thinning was focal in A+T1−, but more extensive in A+T1+ who also showed hippocampal atrophy. At the SCD stage, focal thinning of the EOAD signature, precuneus, and banks of the superior temporal sulcus was observed in A+T1+, whereas no structural alterations were detected in A+T1−. Cortical thinning was associated with episodic and visuospatial memory performance in MCI, while no brain–cognition associations were observed in SCD.

**Conclusion:**

EOAD-related cortical thinning is detectable before dementia and varies across A/T1 profiles, with combined amyloid and *p*-tau181 positivity associated with earlier and more extensive structural involvement.

**Supplementary Information:**

The online version contains supplementary material available at https://doi.org/10.1007/s00415-026-14187-0.

## Introduction

Alzheimer’s disease (AD) is a biologically and clinically heterogeneous neurodegenerative disorder, divided into early-onset (EOAD) and late-onset (LOAD) forms according to whether symptoms emerge before or after the age of 65. Although EOAD accounts for 5–10% of AD cases, it represents the leading cause of degenerative dementia in individuals under 65 years [1, 2]. Its societal and personal impact is disproportionately high, as it affects individuals during their most productive years, causing profound occupational and social disruption [3, 4].

Despite sharing the same pathological hallmarks, extracellular amyloid-β (Aβ) plaques and intracellular neurofibrillary tangles (NFTs), EOAD, and LOAD diverge substantially in their clinical presentation, neuropsychological profiles, and neuroimaging signatures [5–7]. EOAD is associated with faster cognitive decline and an atypical neuropsychological profile compared to LOAD [8].

Data from the Longitudinal Early-Onset Alzheimer’s Disease Study (LEADS) consortium have highlighted a pattern of cortical thinning in EOAD dementia, predominantly involving the inferior lateral temporal cortex, inferior parietal lobule, posterior cingulate, and precuneus, while relatively sparing medial temporal regions. This cortical atrophy pattern has been strongly associated with disease severity, as measured by Clinical Dementia Rating–Sum of Boxes (CDR-SB) and Mini-Mental State Examination (MMSE) scores [9], and has been independently validated in external EOAD cohorts [10]. Evidence from autosomal dominant AD further indicates that cortical alterations may emerge before the onset of overt cognitive impairment. Studies of mutation carriers have demonstrated cortical thinning several years before the expected age of symptom onset, involving regions that partially overlap with the EOAD cortical signature [11, 12]. Notably, structural changes during the earliest preclinical phases may not follow a simple monotonic trajectory, as increased cortical thickness has also been reported in the precuneus and parietotemporal association cortices of presymptomatic PSEN1 mutation carriers [13].

While these findings have contributed to a more refined understanding of the EOAD-specific atrophy signature, several critical gaps remain. First, although structural cortical alterations have been demonstrated before symptom onset in autosomal dominant AD, it remains unclear when the EOAD cortical signature becomes detectable across the early clinical stages of predominantly sporadic, biomarker-confirmed EOAD. In particular, its expression in patients with subjective cognitive decline (SCD) or mild cognitive impairment (MCI) and its relationship with AD biomarker profiles at these stages remain incompletely characterized. This question is particularly relevant given the growing emphasis on early intervention and disease-modifying therapies targeting these stages [14]. Establishing when the EOAD cortical signature first becomes detectable could therefore provide important insights into the earliest structural expression of AD neurodegeneration in younger patients.

Second, it remains unclear whether the emergence of this cortical signature is associated with amyloid positivity alone (A+T1−) or becomes detectable primarily when amyloid positivity is accompanied by an abnormal fluid phosphorylated-tau (*p*-tau) biomarker (A+T1+), reflecting greater biological advancement along the AD continuum [15]. Third, previous work has largely relied on global cognitive screening measures, with limited investigation into how regional cortical atrophy relates to domain-specific cognitive functions in younger individuals.

To address these questions, we first defined the EOAD cortical signature in biomarker-confirmed AD dementia and subsequently examined its expression across biomarker-stratified SCD and MCI groups. Critically, we separated A+T1− from A+T1+ individuals, allowing us to test whether amyloid positivity alone is sufficient for the expression of the EOAD MRI phenotype or whether structural abnormalities become detectable primarily in the presence of an additional abnormal *p*-tau response. We hypothesized that the dementia-stage EOAD cortical signature would show a spatially restricted expression during the preclinical and prodromal phases of AD and that its emergence would be primarily associated with combined A+T1+.

## Methods

### Standard protocol approvals, registrations, and patient consent

This study was conducted in accordance with the Declaration of Helsinki and approved by the Swedish Regional Ethical Review Board. All participants provided written informed consent prior to enrollment. Data and biospecimens were obtained through the Karolinska University Hospital electronic research registry and biobank (GEDOC, Dnr: 2011/1987-31/4, 2020-06484, 2021-04428).

### Participants

Participants were recruited at the Karolinska University Hospital through a collaboration between the Memory Clinic at the Medical Unit Aging, Karolinska University Hospital, and the Division of Clinical Geriatrics, Center for Alzheimer Research at the Department of Neurobiology, Care Sciences, and Society, Karolinska Institutet, Sweden. All individuals underwent a standardized diagnostic protocol comprising general and neurological examinations, clinical history (including informant reports), neuropsychological assessment, 3 T brain MRI, APOE genotyping, and CSF biomarker analysis (Aβ42/40 ratio, phosphorylated tau [*p*-tau181], total tau [*t*-tau], and neurofilament light chain [NfL]).

We included all consecutive patients aged <65 years who had their first clinical visit between April 2018 and May 2024 and met the following inclusion criteria: presence of cognitive complaints and availability of both high-resolution MRI and CSF biomarkers. Exclusion criteria were restricted to unstable medical or psychiatric conditions (e.g., recent cancer, major depression, cardiovascular/cerebrovascular events) or recent initiation of cognitively active treatments. The examination process follows national guidelines established by the Swedish Board of Health and Welfare. A multidisciplinary team evaluates each patient and sets a consensus diagnosis based on all test results. Diagnosis is based on the Diagnostic and Statistical Manual of Mental Disorders (Fifth Edition) (DSM-V) criteria, and International Classification of Diseases, Tenth Revision (ICD-10) coding is used. Patients who do not meet the criteria for MCI or dementia are considered to experience SCD, following the NIA-AA guidelines [16].

A total of 292 patients were included and stratified according to their CSF-based A/T1 biomarker profiles.

The AD continuum group comprised 185 amyloid-positive individuals, including 76 participants with an A+T1− profile and 109 with an A+T1+ profile. The reference group comprised 107 individuals with SCD, negative amyloid and T1 biomarkers (A−T1−), and a low cerebrovascular disease burden, defined as a total Fazekas score <1. Participants who did not meet the criteria for any of these three groups were not included in the present analyses. Amyloid status (A) was determined using the CSF Aβ42/40 ratio, whereas T1 status was defined according to CSF *p*-tau181 concentrations.

### MRI data acquisition, preprocessing, and segmentation

All participants underwent structural brain MRI on a 3T GE Discovery MR750 scanner (GE HealthCare, Milwaukee, WI, USA) using an 8-channel head coil. The protocol included multiple high-resolution sequences: a sagittal 3D T1-weighted BRAVO sequence (1 mm isotropic; TR = 8.16 ms; TE = 3.18 ms), 3D T2-weighted FLAIR CUBE (1.2 mm; TR = 8000 ms; TE = 116 ms), axial T2-weighted PROPELLER (4 mm; TR = 4000 ms; TE = 86 ms), axial diffusion-weighted imaging (DWI; 4 mm; TR = 3501 ms; TE = 62 ms), and axial susceptibility-weighted imaging (SWI 3D; 2 mm; TR = 30 ms; TE = 20 ms).

All scans were visually inspected by experienced neuroradiologists to ensure quality and exclude incidental abnormalities. The Fazekas score for the assessment of white matter (WM) hyperintensities was obtained using the cNeuro cMRI software (Combinostics Oy, Finland, www.cneuro.com/cmri/) [17, 18]. Cortical reconstruction and segmentation were performed using FreeSurfer v7.3.2 (http://surfer.nmr.mgh.harvard.edu/) within the HiveDB neuroinformatics environment [19].

The pipeline included motion correction, skull stripping, Talairach registration, tissue segmentation, and surface generation. Cortical thickness was defined as the distance between the white and pial surfaces. Visual quality control was conducted for all outputs, and all scans passed quality control assessment.

Cortical and subcortical regions were defined using the Desikan–Killiany atlas. Total intracranial volume (TIV) and total WM hypointensity volume were also obtained, with the latter used as a surrogate for cerebrovascular burden [20].

### Definition of the EOAD cortical signature and study design

To define an EOAD-specific pattern of cortical atrophy (hereafter referred to as the “EOAD signature”), we performed a vertex-wise comparison of cortical thickness between patients with CSF-confirmed AD dementia (A+T1− or A+T1+) and A/T1-negative individuals with SCD, who served as the reference group. This analysis was intended to identify cortical regions most characteristically affected in EOAD dementia and aligns with established methods used in prior imaging studies [9, 10].

The EOAD signature was examined both as a mean cortical thickness composite across the retained regions and at the level of the individual Desikan–Killiany parcels. Specifically, cortical thickness within signature regions was examined in AD biomarker-positive individuals at earlier clinical stages (MCI and SCD) to assess the presence and severity of neurodegeneration across the disease continuum.

To evaluate the contribution of an abnormal *p*-tau response beyond amyloid positivity alone, cortical measures were compared between A+T1− and A+T1+ individuals within each clinical stage and compared to the reference group. Finally, within AD biomarker-positive SCD, MCI, and dementia subgroups, we assessed the associations between regional cortical thickness within EOAD signature regions, hippocampal volume, and cognitive performance using domain-specific neuropsychological measures.

### CSF biomarkers and A/T1 classification

CSF samples were collected in polypropylene tubes (Medicarrier, art. no. 67741) and analyzed at the accredited Neurochemistry Laboratory at Karolinska University Hospital. From April 2018 to August 2019, Aβ42, *p*-tau181, and *t*-tau were measured using Innotest ELISA assays (Fujirebio, Ghent, Belgium). From August 2019 onward, analyses were conducted using the Lumipulse G CLEIA platform (Fujirebio Europe), providing higher analytical precision. Cross-platform equivalency has previously been validated [21].

A/T1 classification was based on established cutoffs [22]: Aβ42/40 ratio (× 10) < 0.60 (Innotest) or < 0.86 (Lumipulse), *p*-tau181 ≥ 76 pg/mL (Innotest) or ≥ 56 pg/mL (Lumipulse). CSF total tau (*t*-tau) and neurofilament light chain (NfL) were examined as non-specific markers of neurodegeneration for descriptive purposes.

### Cognitive assessment and APOE genotyping

Cognition was assessed using the Montreal Cognitive Assessment (MoCA) [23] and/or the Mini-Mental State Examination (MMSE) [24]. In a subset, domain-specific functions were further evaluated using: Rey Auditory Verbal Learning Test (RAVLT) for episodic memory [25], Rey-Osterrieth Complex Figure Test (copy and delayed recall) for visual memory and visuospatial processing [26], and Wechsler Adult Intelligence Scale, Fourth Edition (WAIS-IV) Coding subtest for attention and processing speed [27]. The availability of these domain-specific assessments ranged from 54.1% to 63.4% of the cohort (Supplementary Table 1). Depressive symptoms were assessed using the Patient Health Questionnaire-9 (PHQ-9), available in 74.3% of participants [28].

Genetic risk was also assessed through APOE genotyping to determine ε4 carrier status.

### Statistical analysis

Vertex-wise cortical thickness analyses were first performed using a general linear model implemented in FreeSurfer’s Qdec. The EOAD-specific cortical atrophy pattern (the “EOAD signature”) was defined by comparing patients with CSF-confirmed AD dementia to the reference group. Multiple-comparison correction was applied using Monte Carlo simulation (10,000 iterations), with a cluster-forming threshold of *Z* = 3.9 (uncorrected *p* < 0.0001) and a cluster-wise corrected significance threshold of *p* < 0.01. Only clusters with an area ≥500 mm^2^ were retained. When clusters extended across anatomical boundaries, only regions in which at least 33% of the vertices were covered by significant clusters were retained. Analyses were conducted bilaterally, and clusters identified in either hemisphere were included in the final EOAD signature.

Subsequently, clusters defining the EOAD signature were mapped onto Desikan–Killiany parcels and mean cortical thickness values were extracted for each region. These regional measures were then used in group-level analyses. Group comparisons were conducted using analysis of covariance (ANCOVA), adjusting for age and sex. In parallel, bilateral hippocampal volumes were analyzed as a complementary marker of neurodegeneration and disease staging and were normalized for TIV, age, and sex. Total WM hypointensity volumes were normalized for TIV.

To characterize stage-dependent expression of the EOAD signature, group-wise differences in cortical thickness within the signature regions and hippocampal volume were examined across the clinical continuum (SCD, MCI, and AD dementia) among AD biomarker-positive individuals.

Within SCD and MCI stages, associations between APOE ε4 carrier status and biomarker group (A+T1− vs. A+T1+) were assessed using chi-square (*χ*^2^) tests to determine whether differences in *p*-tau181 status among amyloid-positive individuals were accompanied by differences in APOE-related genetic risk. Finally, within the biomarker-defined AD continuum, associations between regional brain atrophy and cognitive performance were explored using Pearson’s partial correlation analyses adjusted for age, sex, and education. Because extended neuropsychological measures are susceptible to floor effects in dementia, analyses in this subgroup were restricted to global cognitive measures.

False discovery rate (FDR) correction was applied to account for multiple comparisons, and Bonferroni-adjusted pairwise post hoc tests were used where appropriate. Statistical significance was set at *p* < 0.05 (two tailed). Cohen’s *d* effect sizes were calculated to estimate the magnitude of pairwise group differences. All statistical analyses were conducted using R (version 4.4.2).

## Results

### Study population characteristics

The study sample comprised 292 participants (mean age 58.0 ± 5.1 years; 61.8% female). Most participants presented with SCD (*n* = 149, 50.7%; mean age 56.7 ± 5.4 years), followed by MCI (*n* = 76, 26.0%; mean age 59.2 ± 4.7 years) and dementia (*n* = 68, 23.3%; mean age 59.3 ± 3.8 years) (Table 1). One-hundred-seven participants were classified as the reference group. Seventy-six participants had isolated amyloid positivity (A+T1−), whereas 109 had both amyloid and CSF *p*-tau181 positivity (A+T1+). Within the A+T1− group, 26 participants had SCD, 37 had MCI, and 13 had dementia. Within the A+T1+ group, 15 participants had SCD, 39 had MCI, and 55 had dementia. Overall, the biomarker-amyloid-positive sample comprised 41 participants with SCD, 76 with MCI, and 68 with dementia.

**Table 1:** Summary of demographic characteristics, cognitive assessment, cerebrovascular burden, and CSF biomarkers across A/T1 profiles

	Available *n*	All (*n* = 292)	Controls (*n* = 107)	A+T1− (*n* = 76)	A+T1+ (*n* = 109)	Global *p*-value and pairwise comparisons
Demographic variables						
Age (years)	292	58.0 (5.1)	56.6 (5.5)	58.2 (5.2)	59.1 (4.2)	0.002^b^
Gender (female, %)	292	61.8%	63.0%	57.9%	63.3%	0.720
Education (years)	230	13.3 (3.4)	13.8 (3.4)	13.5 (3.8)	12.7 (3.2)	0.134
APOE (ε4 +/− or +/+, %)	284	56.7%	37.5%	66.7%	68.6%	<0.001^a,b^
Cognitive assessment						
MoCA	233	23.1 (5.3)	26.8 (1.9)	22.3 (5.3)	20.2 (5.5)	<0.001^a,b^
MMSE	188	26.2 (4.0)	28.6 (1.4)	25.7 (4.4)	24.5 (4.3)	<0.001^a,b^
RAVLT	185	41.8 (13.4)	49.7 (9.4)	39.9 (12.6)	34.8 (13.5)	<0.001^a,b^
RAVLT delayed recall	183	8.8 (4.5)	11.5 (3.0)	7.9 (4.0)	6.5 (4.7)	<0.001^a,b^
RCF copy	158	31.2 (6.5)	32.9 (3.2)	30.5 (7.0)	29.9 (8.4)	0.038^b^
RCF delayed recall	179	15.2 (8.3)	19.7 (7.2)	14.2 (6.7)	10.8 (8.3)	<0.001^a,b^
WAIS-IV coding	180	51.7 (17.0)	58.5 (12.6)	50.2 (18.5)	45.2 (17.4)	<0.001^a,b^
PHQ-9	217	7.3 (5.7)	8.4 (5.6)	7.4 (6.3)	6.2 (5.2)	0.065^b^
CSF biomarkers						
Aβ42/Aβ40	292	0.07 (0.03)	0.10 (0.01)	0.07 (0.01)	0.05 (0.01)	<0.001^a,b,c^
*p*-tau181, pg/ml	292	63.7 (42.5)	32.9 (9.3)	47.4 (10.3)	105.5 (42.7)	<0.001^a,b,c^
*t*-tau, pg/ml	292	405.3 (249.6)	230.9 (76.0)	306.0 (87.7)	647.3 (246.8)	<0.001^a,b,c^
NfL, pg/ml	292	935.7 (604.7)	671.1 (211.0)	1006.4 (937.7)	1148.6 (463.3)	<0.001^a,b^
WM hypointensities						
WM hypointensities (mm^3^)	292	2414.6 (5279.9)	1333.7 (5089.4)	3042.8 (6880.8)	3047.6 (3870.5)	0.004^a,b^
Clinical stage						
SCD	292	50.7%	100.0%	34.2%	13.8%	<0.001^a,b,c^
MCI		26.0%	0.0%	48.7%	35.8%	
Dementia		23.3%	0.0%	17.1%	50.5%	

Quantitative data are presented as mean (SD); categorical data as percentages. Available n indicates the number of non-missing observations included for each variable. Global *p*-values were obtained using one-way ANOVA for quantitative variables and χ^2^ tests for categorical variables, then corrected for false discovery rate (FDR). Letters indicate significant pairwise comparisons after Bonferroni correction: a Controls vs. A+T1−; b Controls vs. A+T1+; c A+T1− vs. A+T1+. Analyses used available observations for each variable

WM hypointensities are presented as raw data; group comparisons were performed after normalization to estimated total intracranial volume (TIV)

*APOE* apolipoprotein E, MoCA Montreal Cognitive Assessment, *MMSE* Mini-Mental State Examination, *RAVLT* Rey Auditory Verbal Learning Test, *RCF* Rey Complex Figure Test, *WAIS* Wechsler Adult Intelligence Scale, *PHQ*-*9* Patient Health Questionnaire, *WM* white matter, *Aβ* amyloid-beta, *p*-*tau181* phosphorylated tau, *t-tau* total tau, *NfL* neurofilament light chain, *SCD* subjective cognitive decline, *MCI* mild cognitive impairment, *TIV* estimated total intracranial volume

### Cortical signature of EOAD

**Fig. 1:**
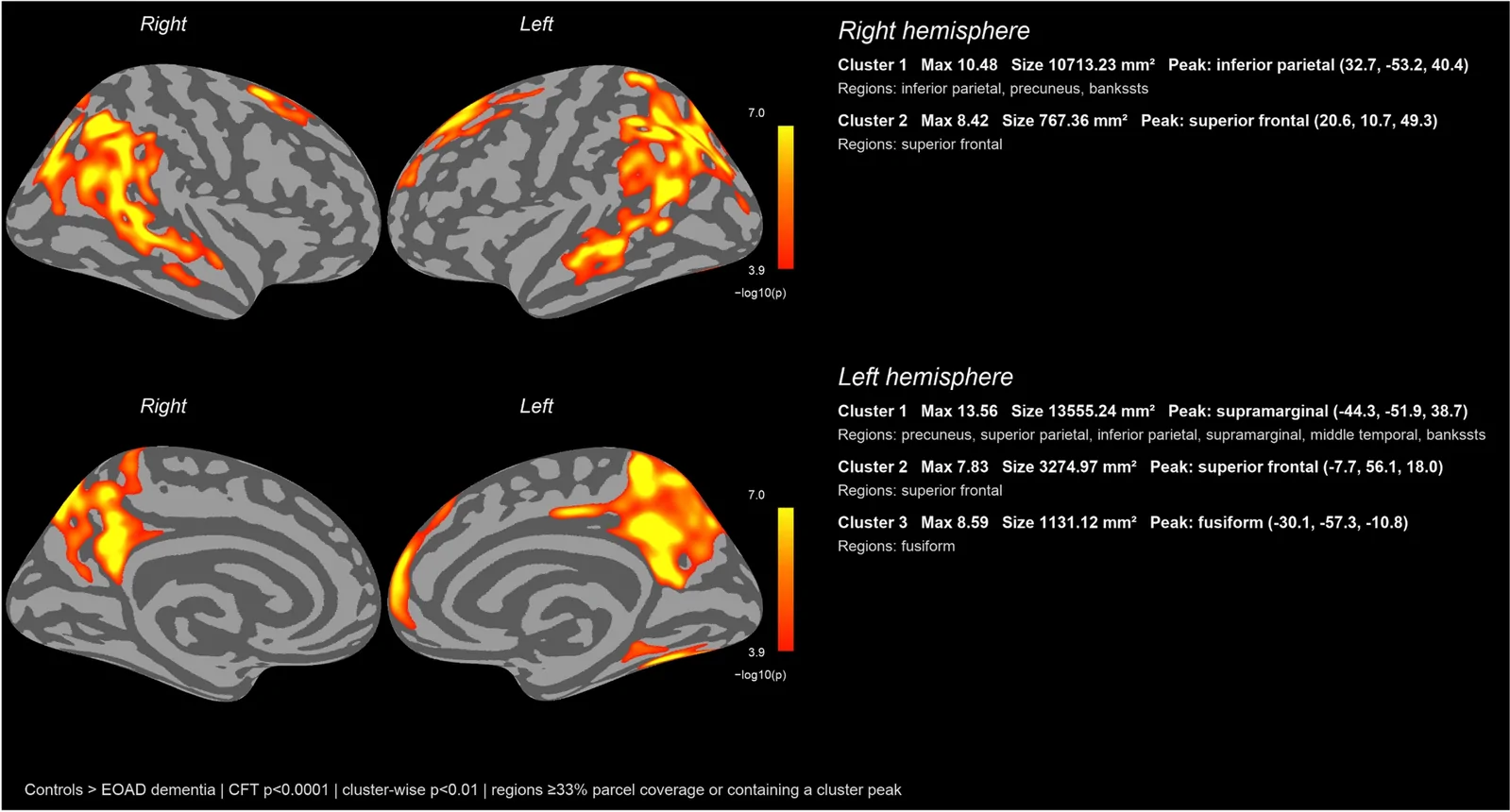
Cortical thinning pattern in EOAD dementia compared to the reference group of A−/T1− SCD. Vertex-wise cortical thickness analysis revealed significant atrophy in EOAD dementia patients relative to the reference group, using Monte Carlo simulation with 10,000 iterations, a cluster-forming threshold of *Z* = 3.9 (uncorrected *p* < 0.0001), and a cluster-wise corrected significance threshold of *p* < 0.01. The table reports, for each cluster, the maximum *Z*-score (Max), the cluster size in mm^2^, and the Talairach coordinates (*X*, *Y*, *Z*) of the peak vertex

Whole-cortex vertex-wise analysis in patients with AD dementia revealed a robust and spatially coherent pattern of cortical thinning relative to biomarker-negative SCD individuals (Fig. 1). The most severely affected regions were located within the bilateral posterior parietal and lateral association cortices, particularly the precuneus, inferior and superior parietal lobules, and supramarginal gyrus. Additional atrophic clusters extended into the banks of the superior temporal sulcus bilaterally and the superior frontal, middle temporal, and fusiform gyri, with a predominance in the left hemisphere. This pattern defined the EOAD cortical signature used for all subsequent analyses.

### Structural changes along the AD continuum

**Fig. 2:**
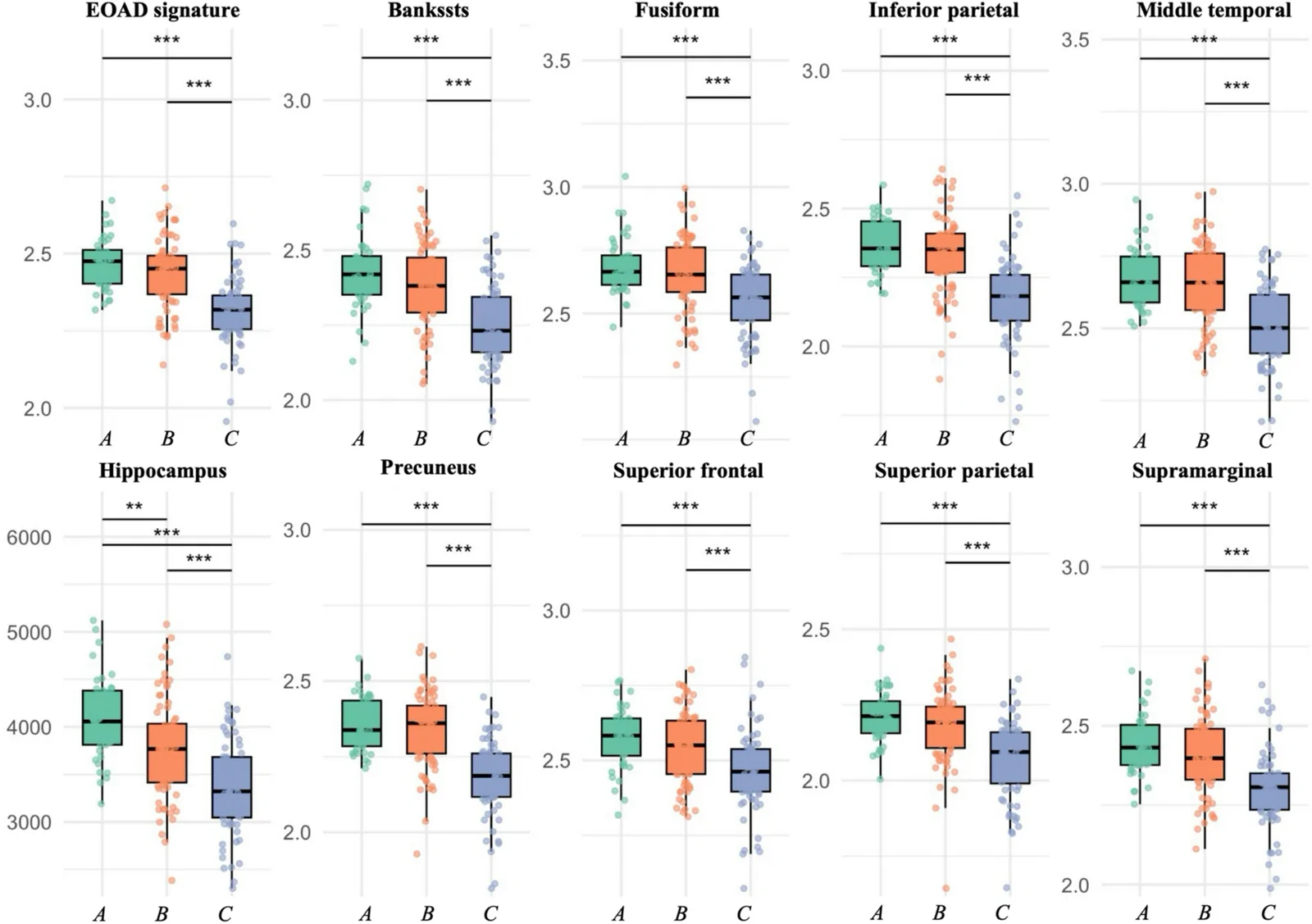
Cortical thickness and hippocampal volume across the AD continuum. Boxplots show group-wise differences in cortical thickness (mm) and hippocampal volume (mm^3^) across three clinical stages of the AD continuum. Group definitions: A—SCD AD (*n* = 41), B—MCI-AD (n = 76), C—AD dementia (*n* = 68). All metrics are reported as mean cortical thickness (mm) except for the hippocampus, which is represented as volume (mm^3^) and was normalized for TIV in ANCOVA models. Statistical comparisons were performed using ANCOVA models adjusted for age and sex, followed by Bonferroni-corrected post hoc tests. Asterisks denote statistical significance: *p < 0.05; **p < 0.01; ***p < 0.001. *Bankssts* banks of the superior temporal sulcus

Patients with AD dementia showed the greatest cortical thinning across all EOAD signature regions, whereas SCD and MCI exhibited comparable cortical thickness. In contrast, hippocampal volume was significantly reduced in MCI compared to SCD (*t* = –3.51, *p* = 0.0017, *d* = –0.71) (Fig. 2).

### Cortical thickness and hippocampal volume in MCI

Marked cortical thinning and hippocampal volume loss were observed in MCI individuals with biomarker-confirmed AD (MCI-AD) compared to the reference group. A+T1− MCI individuals showed lower cortical thickness in the EOAD signature (*t* = −2.60, *p* = 0.0303, *d* = −0.51), banks of the superior temporal sulcus (*t* = −3.24, *p* = 0.0043, *d* = −0.63), middle temporal gyrus (*t* = −2.45, *p* = 0.0459, *d* = −0.48), and supramarginal gyrus (*t* = −2.77, *p* = 0.0184, *d* = −0.54), without a significant reduction in hippocampal volume. A+T1+ MCI individuals exhibited a more extensive pattern of cortical thinning, encompassing the EOAD signature (*t* = −4.41, *p* < 0.001, *d* = −0.84), banks of the superior temporal sulcus (*t* = −3.93, *p* < 0.001, *d* = −0.75), fusiform gyrus (*t* = −3.07, *p* = 0.0075, *d* = −0.59), inferior parietal lobule (*t* = −4.60, *p* < 0.001, *d* = −0.88), middle temporal gyrus (*t* = −4.09, *p* < 0.001, *d* = −0.78), precuneus (*t* = −3.74, *p* < 0.001, *d* = −0.71), superior frontal gyrus (*t* = −2.73, *p* = 0.0211, *d* = −0.52), superior parietal lobule (*t* = −3.46, *p* = 0.0020, *d* = −0.66), and supramarginal gyrus (*t* = −3.72, *p* < 0.001, *d* = −0.71). Hippocampal volume was also markedly lower in A+T1+ MCI individuals (*t* = −5.23, *p* < 0.001, *d* = −1.00).

**Fig. 3:**
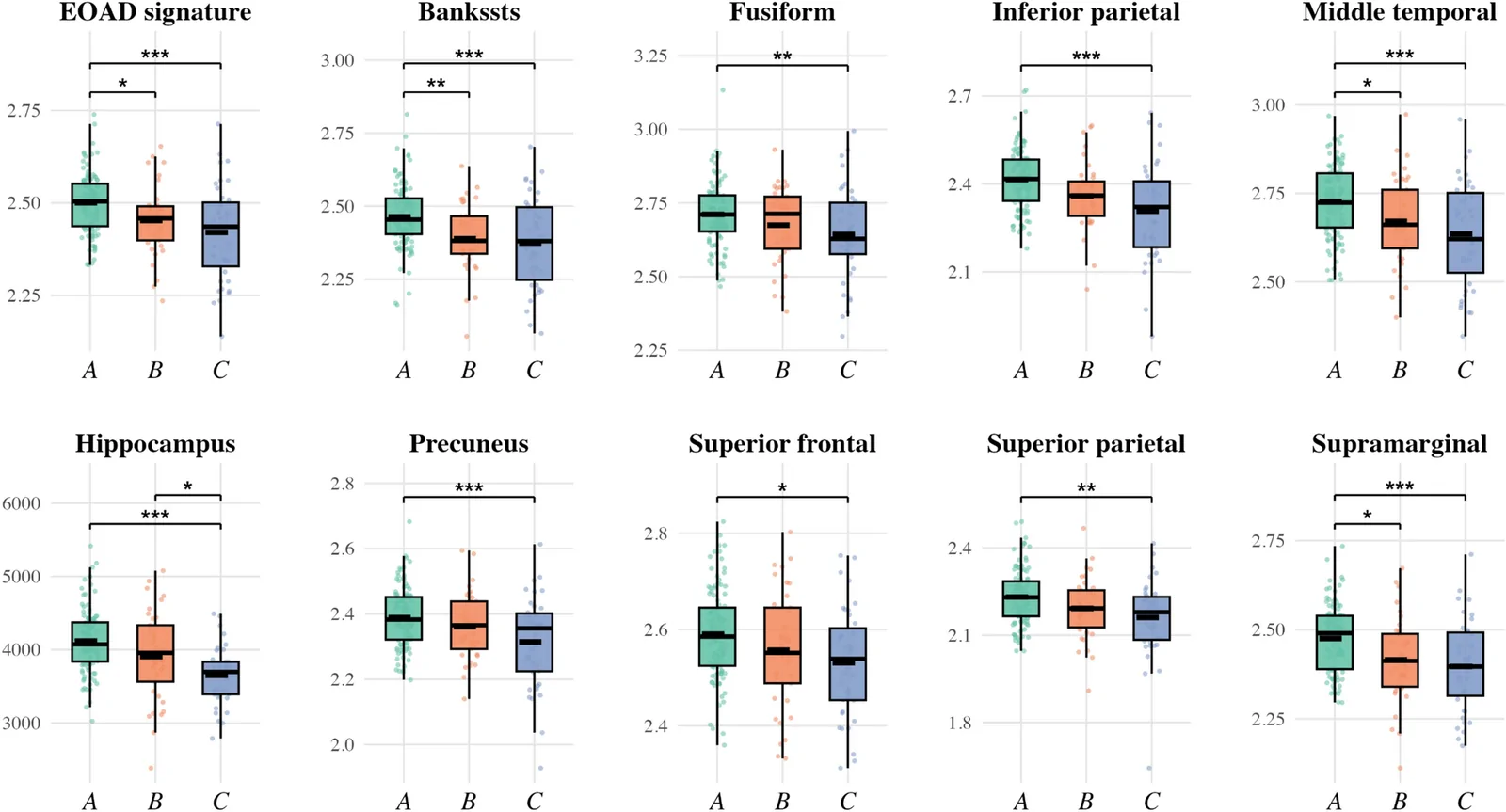
Regional cortical thickness and hippocampal volume across MCI A/T1 biomarker profiles. Boxplots show differences in mean cortical thickness within the EOAD signature and individual signature regions, and in hippocampal volume, across the reference group and MCI subgroups stratified by A/T1 biomarker status. Group definitions: A—Reference group: SCD with negative amyloid and T1 biomarkers (A−T1−; *n* = 107); B—MCI with amyloid positivity and negative T1 biomarker (A+T1−; *n* = 37); C—MCI with amyloid and T1 biomarker positivity (A+T1+; *n* = 39). Cortical measures are expressed as mean cortical thickness (mm), whereas the hippocampus is reported as volume (mm^3^). Group differences were assessed using ANCOVA models adjusted for age and sex; hippocampal volume was additionally normalized for total intracranial volume (TIV). Pairwise post hoc comparisons were Bonferroni corrected. Asterisks denote statistical significance: *p < 0.05; **p < 0.01; ***p < 0.001. *Bankssts* banks of the superior temporal sulcus, *SCD* subjective cognitive decline, *MCI* mild cognitive impairment, *TIV* total intracranial volume

Direct comparison of the two MCI biomarker groups showed lower hippocampal volume in A+T1+ than in A+T1− individuals (*t* = −2.46, *p* = 0.0450, *d* = −0.56), whereas none of the cortical thickness measures differed significantly after Bonferroni correction (Fig. 3).

### Cortical thickness and hippocampal volume in SCD

**Fig. 4:**
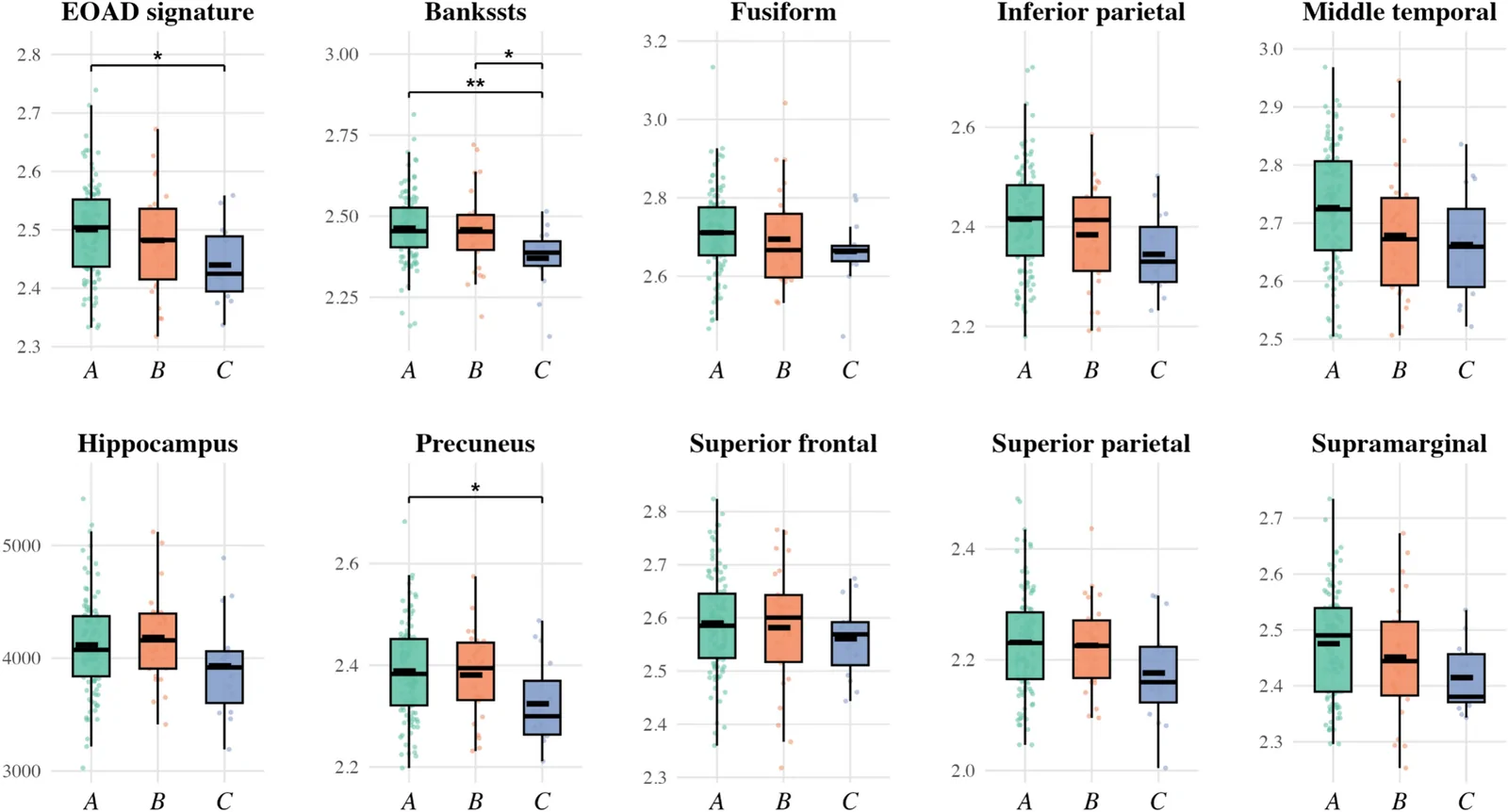
Regional cortical thickness and hippocampal volume across SCD A/T1 biomarker profiles. Boxplots show differences in mean cortical thickness within the EOAD signature and individual signature regions, and in hippocampal volume, across SCD groups stratified by A/T1 biomarker status. Group definitions: A—Reference group: SCD with negative amyloid and T1 biomarkers (A−T1−; *n* = 107); B—SCD with amyloid positivity and negative T1 biomarker (A+T1−; *n* = 26); C—SCD with amyloid and T1 biomarker positivity (A+T1+; *n* = 15). Cortical measures are expressed as mean cortical thickness (mm), whereas the hippocampus is reported as volume (mm^3^). Group differences were assessed using ANCOVA models adjusted for age and sex; hippocampal volume was additionally normalized for total intracranial volume (TIV). Pairwise post hoc comparisons were Bonferroni corrected. Asterisks denote statistical significance: *p < 0.05; **p < 0.01; ***p < 0.001. *Bankssts*, banks of the superior temporal sulcus, *SCD* subjective cognitive decline, *TIV* total intracranial volume

No global group effect survived FDR correction across cortical thickness measures or hippocampal volume. Nevertheless, Bonferroni-adjusted pairwise comparisons identified focal cortical thinning in A+T1+ SCD individuals relative to the reference group, involving the EOAD signature (*t* = −2.69, *p* = 0.0242, *d* = −0.75), banks of the superior temporal sulcus (*t* = −3.13, *p* = 0.0064, *d* = −0.87), and precuneus (*t* = −2.45, *p* = 0.0464, *d* = −0.68). A+T1+ SCD individuals also showed lower cortical thickness in the banks of the superior temporal sulcus compared with A+T1− SCD individuals (*t* = −2.51, *p* = 0.0391, *d* = −0.83). No significant cortical alterations were observed in A+T1− SCD individuals relative to the reference group, and hippocampal volume did not differ between groups (Fig. 4).

### Brain–cognition associations in AD biomarker-positive SCD, MCI, and dementia

**Fig. 5:**
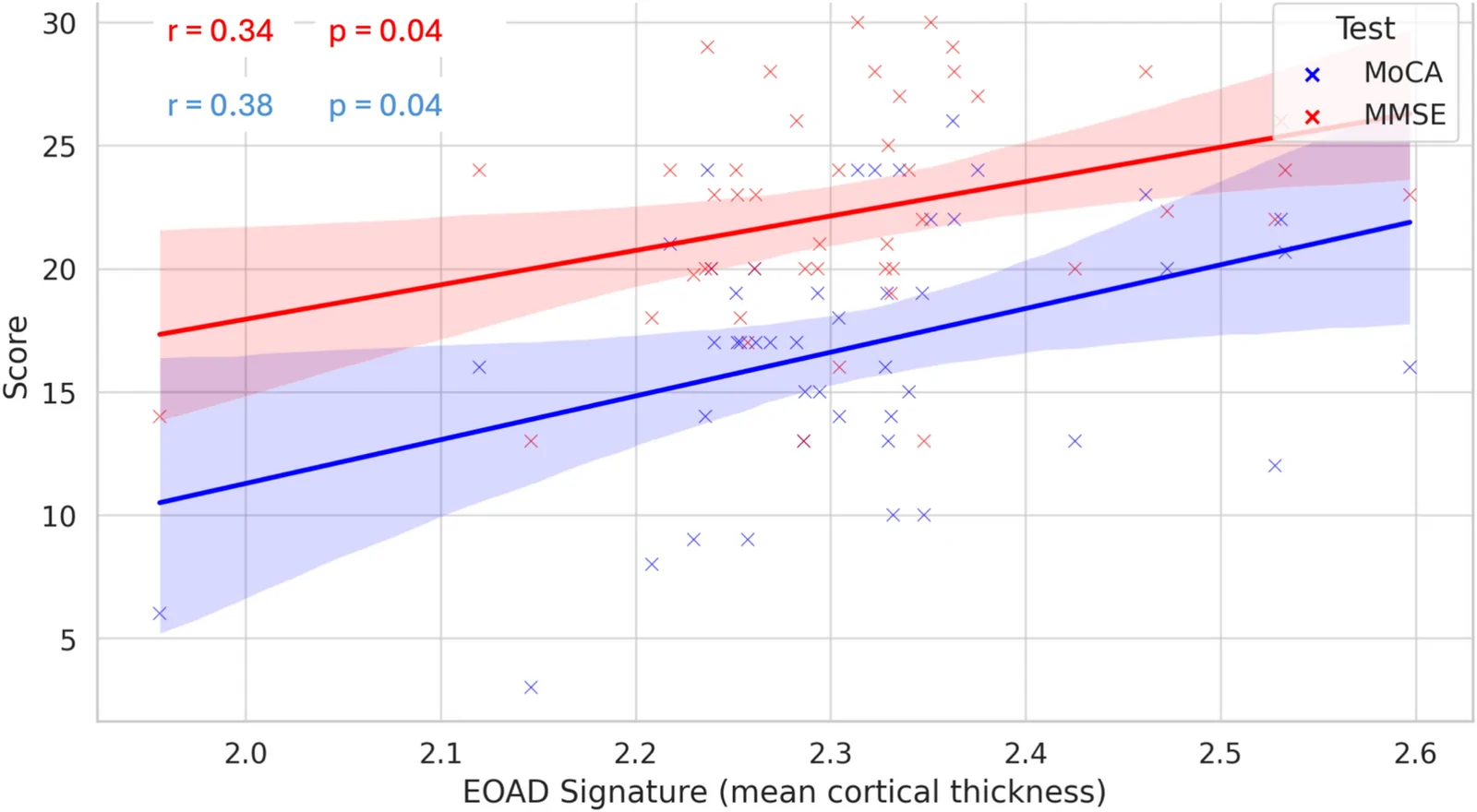
EOAD signature and global cognition in patients with AD dementia. Scatterplots illustrating Pearson correlations between mean cortical thickness in the EOAD signature and global cognition in patients with AD dementia. Red indicates MMSE scores (*r* = 0.34), blue indicates MoCA scores (*r* = 0.38). Solid lines represent linear regression fits with shaded 95% confidence intervals

Among patients with AD biomarker positivity, specific associations emerged between regional cortical atrophy and cognitive performance. In the dementia group, significant correlations were observed between MMSE and/or MoCA scores and cortical thinning in the EOAD signature composite (*r* > 0.30, *p* < 0.05; Fig. 5).

**Fig. 6:**
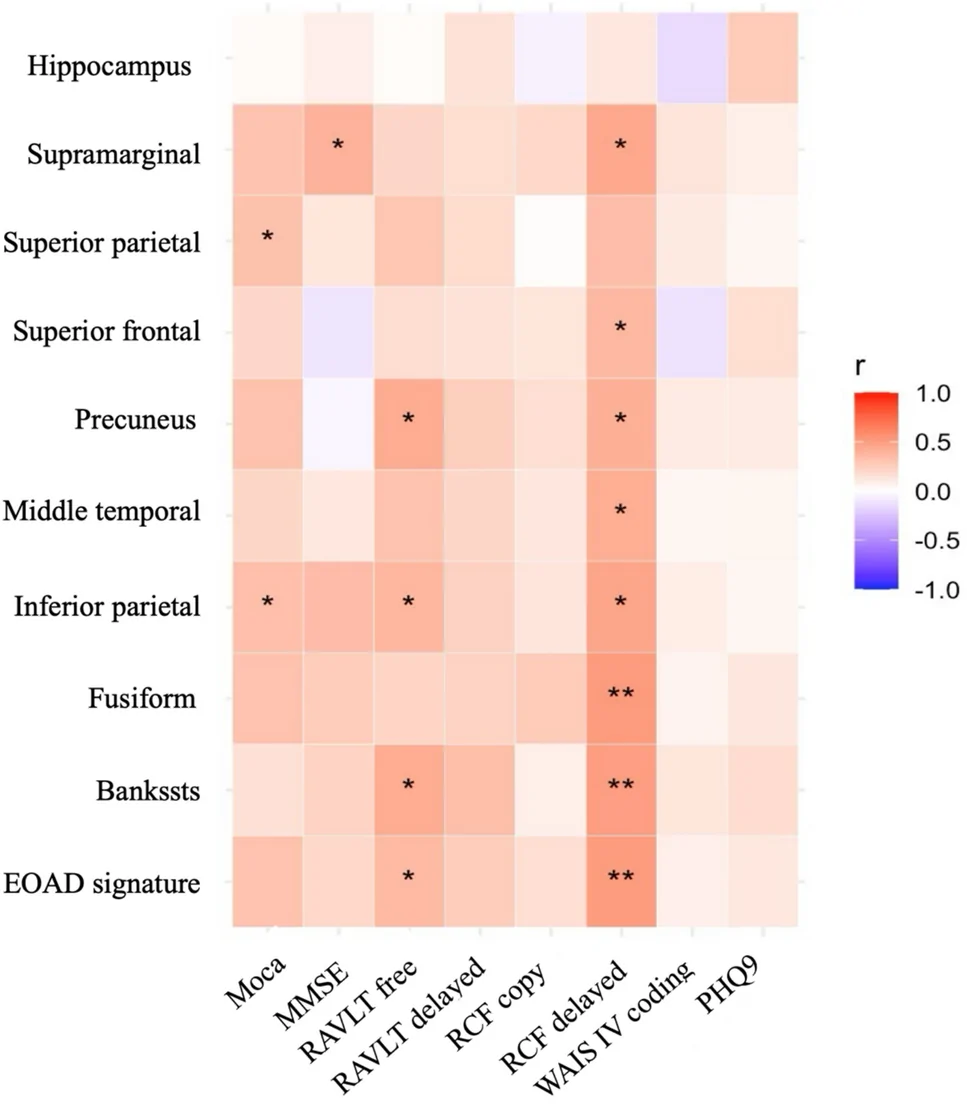
Associations between regional atrophy and cognitive performance in MCI-AD biomarker-positive (A+T1− or A+T1 +) patients. Heatmap showing partial correlations (adjusted for age, sex, and education) between cortical thickness or hippocampal volume and cognitive test scores in MCI with AD biomarkers. The strength and direction of associations are represented by the color gradient, with positive correlations in red and negative correlations in blue. Cognitive domains are assessed using the following: MoCA: global cognition, MMSE: global cognition, RAVLT free / RAVLT delayed: episodic memory, RCF copy / RCF delayed: visuospatial construction and memory, WAIS-IV coding: Processing speed and attention, PHQ-9: Depressive symptoms. All imaging variables are in cortical thickness (mm), except for the hippocampus, which is expressed as volume (mm^3^) and normalized for TIV. Significance levels are denoted as follows: *p < 0.05; **p < 0.01, after FDR correction. Only statistically significant associations are marked with asterisks. *Bankssts* banks of the superior temporal sulcus, *MoCA* Montreal Cognitive Assessment, *MMSE* Mini-Mental State Examination, *RAVLT* Rey Auditory Verbal Learning Test, *RCF* Rey-Osterrieth Complex Figure, *WAIS-IV* Wechsler Adult Intelligence Scale, Fourth Edition, *PHQ-9* Patient Health Questionnaire-9

In the MCI-AD subgroup, cortical thinning in the inferior parietal cortex was correlated with lower MoCA scores (*r* = 0.33, *p* = 0.048). Lower thickness in the precuneus and supramarginal gyrus was associated with poorer performance in recall (*r* = 0.32–0.33, *p* < 0.05). Moreover, thinner cortex in the EOAD signature, fusiform gyrus, and banks of the superior temporal sulcus was significantly associated with lower visuospatial memory as measured by the RCF delayed recall (*r* = 0.45–0.47, *p* < 0.01) (Fig. 6).

No significant correlations between regional atrophy and cognitive performance were found among SCD individuals with AD biomarkers.

### APOE ε4 differences between A+T1- and A+T1+ groups

APOE ε4 carrier status was not associated with biomarker group across clinical stages. In the SCD group, APOE ε4 frequency did not differ between A+T1− and A+T1+ (*χ*^2^ = 0.51, *p* = 0.48). Likewise, in the MCI group, no difference in APOE ε4 carriage was observed between biomarker groups (*χ*^2^ = 0.03, *p* = 0.87).

## Discussion

This study extends previous work on the EOAD imaging phenotype by characterizing its expression across predementia clinical stages and A/T1 biomarker profiles in a predominantly sporadic, memory clinic population. Cortical thinning before overt dementia has previously been demonstrated in familial AD mutation carriers [11, 12]. Our findings complement this evidence by showing that the cortical pattern described in sporadic EOAD dementia can also be detected, to varying degrees, in biomarker-confirmed SCD and MCI. Importantly, its expression followed a biomarker-dependent gradient, being undetectable in A+T1− SCD, focal in A+T1+ SCD, and more extensively expressed in A+T1+ MCI. Regional cortical thinning was also associated with domain-specific cognitive performance at the MCI stage.

In line with prior large-scale studies, including data from the LEADS consortium [9], we identified a robust and spatially consistent EOAD atrophy signature in patients with AD dementia, characterized by cortical thinning in the precuneus, superior and inferior parietal lobules, supramarginal gyrus, banks of the superior temporal sulcus, fusiform, middle temporal, and superior frontal regions. This distribution closely mirrors previously described EOAD-specific patterns, supporting its external validity and biological relevance across independent cohorts. The observed left-hemispheric predominance (Fig. 1) further aligns with prior reports in younger populations, reinforcing the hypothesis that EOAD represents a distinct neurodegenerative phenotype with specific spatial and possibly molecular underpinnings [9, 10].

Crucially, this atrophy pattern was not confined to the dementia stage. In MCI with AD biomarkers, we observed significant cortical thinning across most EOAD-related regions, alongside marked hippocampal atrophy. These findings indicate that widespread neocortical neurodegeneration may already be underway at the prodromal stage. Moreover, stratification by *p*-tau181 status showed that a more advanced biomarker profile within the AD continuum was associated with greater structural brain changes. A+T1+ individuals exhibited the most extensive cortical thinning and hippocampal atrophy, consistent with converging evidence [29–31]. Among A+T1− MCI individuals, the banks of the superior temporal sulcus showed the largest effect relative to the A/T1-negative SCD reference group. This region has been previously identified as an early site of amyloid accumulation in amyloid-PET studies [32] and is among the first to exhibit cortical thinning in autosomal dominant AD [33]. Its early involvement before detectable *p*-tau181 positivity may reflect a particular vulnerability to amyloid-induced synaptic dysfunction.

Notably, hippocampal volume was the only structural measure that significantly differed between A+T1+ and A+T1− individuals at the MCI stage. Hippocampal atrophy is well recognized in EOAD, despite the relatively greater neocortical involvement generally observed compared with LOAD. In our cohort, A+T1− individuals showed focal cortical thinning relative to the reference group without significant hippocampal volume loss, whereas A+T1+ individuals exhibited both more extensive neocortical thinning and marked hippocampal atrophy. These findings suggest that hippocampal volume may complement the EOAD cortical signature in characterizing structural disease burden at the prodromal stage, particularly in individuals with a more advanced biomarker profile.

A particularly relevant observation was the detection of focal EOAD signature thinning in A+T1+ individuals who had not yet developed objective cognitive impairment. Specifically, A+T1+ SCD individuals showed subtle but significant cortical thinning in EOAD-relevant regions, particularly the precuneus and banks of the superior temporal sulcus, whereas no significant alterations were detected in A+T1− SCD individuals. These findings are consistent with evidence from autosomal dominant AD showing that cortical thinning can precede symptom onset by several years [11]. Notably, the precuneus emerged as an early site of structural change in that setting and was also among the few regions affected in our A+T1+ SCD group, suggesting that posterior association cortices may represent sites of early structural vulnerability across both familial and predominantly sporadic young-onset AD. Our findings extend this evidence by providing a biomarker-based perspective on these early structural changes, showing that, within SCD, MRI-detectable cortical abnormalities were observed in A+T1+ but not A+T1− individuals.

CSF *p*-tau181 should not, however, be interpreted as a direct marker of neurofibrillary tau pathology; under the revised biomarker framework, it represents a T1 biomarker that may reflect an amyloid-associated response linking Aβ pathology to subsequent tau-related changes. Accordingly, the present findings suggest that MRI-detectable cortical abnormalities in SCD individuals are associated with a more advanced biomarker profile within the AD continuum [15, 34–36]. The absence of cortical thinning in A+T1− SCD cases may thus reflect a temporal dissociation between amyloid accumulation and subsequent neurodegenerative processes. Two complementary hypotheses may explain the limited atrophy observed in A+T1− individuals. First, this pattern may reflect an earlier stage of the AD continuum, with A+T1− individuals subsequently progressing to A+T1+, as suggested by longitudinal A/T conversion studies [37, 38]. Consistent with this interpretation, in our cohort A+T1− individuals were more frequently observed in earlier clinical stages, being more prevalent in SCD (34.2%) and MCI (48.6%), whereas A+T1+ individuals were proportionally more represented in MCI (35.7%) than in SCD (13.7%). Second, A+T1− and A+T1+ may delineate biologically distinct subtypes of AD, with differential rates of atrophy and clinical progression. Indeed, A+T1− MCI cases, often characterized by minimal or absent atrophy, have been associated with a slower progression and a more indolent subtype of AD, whereas A+T1+ individuals showed a significantly higher risk of conversion within a few years [39]. An additional, although speculative, explanation is that early amyloid-related processes may be associated with transient increases in cortical thickness or volume, potentially masking subtle neurodegenerative changes in A+T1− individuals, as previously suggested in presymptomatic PSEN1 mutation carriers [13].

Notably, APOE ε4 carriage did not differ between A+T1− and A+T1+ individuals within either the SCD or MCI stage, suggesting that the observed biomarker-dependent structural differences were not accompanied by systematic differences in APOE ε4 frequency.

We also observed increasing neurodegeneration across the AD continuum, with the greatest cortical involvement in dementia and earlier hippocampal volume loss at the MCI stage. This finding corroborates our recent systematic review, which demonstrated that hippocampal subfields, hence overall hippocampal volume, show their steepest decline at the transition from SCD to MCI, marking the clinical inflection point of AD progression [40].

Correlation analyses highlighted the functional significance of the EOAD atrophy signature. In AD dementia, lower thickness within this signature correlated with global cognitive performance on MMSE and MoCA, in line with recent reports [9, 10]. In MCI-AD, thinner inferior parietal cortex and precuneus were associated with poorer episodic memory, while fusiform and banks of the superior temporal sulcus involvement were related to impaired visuospatial memory. No such associations were observed in SCD, suggesting that structural–cognitive coupling emerges only once a critical threshold of neurodegeneration is reached, as recently hypothesized [41], particularly in regions central to the EOAD phenotype.

Our findings have direct translational relevance. By defining the stage- and biomarker-dependent emergence of the EOAD cortical signature, we show that regional cortical thickness in EOAD-relevant areas can identify biologically active neurodegeneration in younger individuals. These findings support regional cortical thickness as a candidate MRI staging marker for biologically defined EOAD in younger memory clinic patients. Notably, A+T1+ individuals at the SCD stage appear to represent a clinically silent yet biologically progressive phase of AD, supporting their prioritization for closer surveillance and inclusion in secondary prevention trials.

This study has limitations. The cross-sectional design precludes causal inference regarding individual disease trajectories. While the cohort is one of the largest biomarker-stratified EOAD samples in a real-world memory clinic setting to date, findings may not generalize to population-based or non-referral settings. Conversely, this setting enhances the clinical relevance and translational value of our findings by reflecting the heterogeneity of younger patients undergoing diagnostic evaluation for cognitive concerns in routine memory clinic practice. Cognitive characterization was also limited by the availability of comprehensive neuropsychological testing in only a subset of participants. Although clinical diagnoses were established through multidisciplinary assessment, the available cognitive battery may not have fully captured the heterogeneous and multidomain cognitive profiles characteristic of EOAD [42]. Moreover, the considerable scatter and the modest proportion of variance explained by MMSE and MoCA indicate that these global screening measures capture only a limited component of the variability in EOAD-related cortical thinning. A further limitation of the present study is the lack of comprehensive genetic testing for genes classically associated with monogenic AD (PSEN1, PSEN2, and APP), which precluded estimation of the proportion of monogenic AD cases in our cohort. Notably, APOE genotyping was systematically available, allowing us to account for the major genetic risk factor for sporadic AD [43]. In addition, the absence of an independent external validation cohort should be acknowledged. Because the EOAD signature was derived in the dementia subgroup and interrogated in earlier-stage subgroups from the same clinical cohort, the analyses should be considered an internal stage-extension rather than an independent validation. The study therefore establishes the plausibility and biomarker dependence of predementia EOAD signature expression, but external replication is required before the regional pattern can be considered generalizable or diagnostically applicable.

In individuals younger than 65 years, the established EOAD dementia signature can be traced backward into earlier clinical stages, but its expression follows a marked biomarker gradient. Isolated amyloid positivity was associated with little or no detectable cortical thinning, whereas combined amyloid and *p*-tau181 positivity was linked to focal EOAD-pattern involvement in SCD and widespread involvement in MCI. These findings identify the biomarker conditions under which the EOAD cortical phenotype becomes MRI-detectable before dementia and provide a foundation for longitudinal validation of cortical thickness as a staging marker in biologically defined EOAD.

## Supplementary Information

Below is the link to the electronic supplementary material.

Supplementary file1 (DOCX 29 KB)

## Data Availability

We are open to requests for data collected in this study. Proposals must include a detailed study plan outlining the research question, analysis plan, and specific data needed. Only deidentified data and the data dictionary are shared. Analyses will be conducted in collaboration with our team, and data access will be subject to the GEDOC legal framework. A formal data access agreement will be needed.

## References

[1] Rossor MN, Fox NC, Mummery CJ et al (2010) The diagnosis of young-onset dementia. Lancet Neurol 9:793–806. 10.1016/S1474-4422(10)70159-9 PMC2947856 20650401

[2] Ayodele T, Rogaeva E, Kurup JT et al (2021) Early-onset Alzheimer’s disease: what is missing in research? Curr Neurol Neurosci Rep 21:4. 10.1007/s11910-020-01090-y PMC7815616 33464407

[3] van Vliet D, de Vugt ME, Bakker C et al (2010) Impact of early onset dementia on caregivers: a review. Int J Geriatr Psychiatry 25:1091–1100. 10.1002/gps.2439 20957692

[4] Hendriks S, Peetoom K, Bakker C et al (2021) Global prevalence of young-onset dementia: a systematic review and meta-analysis. JAMA Neurol 78:1080–1090. 10.1001/jamaneurol.2021.2161 PMC8290331 34279544

[5] Reitz C, Rogaeva E, Beecham GW (2020) Late-onset vs nonmendelian early-onset Alzheimer disease. Neurol Genet 6:e512. 10.1212/NXG.0000000000000512 PMC7673282 33225065

[6] van der Flier WM, Pijnenburg YA, Fox NC, Scheltens P (2011) Early-onset versus late-onset Alzheimer’s disease: the case of the missing APOE ɛ4 allele. Lancet Neurol 10:280–288. 10.1016/S1474-4422(10)70306-9 21185234

[7] Mendez MF (2019) Early-onset Alzheimer disease and its variants. Continuum (Minneap Minn) 25:34–51. 10.1212/CON.0000000000000687 PMC6538053 30707186

[8] Tort‐Merino A, Falgàs N, Allen IE et al (2022) Early‐onset Alzheimer’s disease shows a distinct neuropsychological profile and more aggressive trajectories of cognitive decline than late‐onset. Ann Clin Transl Neurol 9:1962–1973. 10.1002/acn3.51689 PMC9735361 36398437

[9] Touroutoglou A, Katsumi Y, Brickhouse M et al (2023) The sporadic early-onset Alzheimer’s disease signature of atrophy: preliminary findings from the Longitudinal Early-onset Alzheimer’s Disease Study (LEADS) cohort. Alzheimers Dement 19(Suppl 9):S74–S88. 10.1002/alz.13466 PMC10829523 37850549

[10] Mehta RI, Keith CM, Teixeira CVL et al (2024) The early-onset Alzheimer’s disease MRI signature: a replication and extension analysis in early-stage AD. Cereb Cortex 34:bhae475. 10.1093/cercor/bhae475 PMC11664631 39714256

[11] Weston PSJ, Nicholas JM, Lehmann M et al (2016) Presymptomatic cortical thinning in familial Alzheimer disease: a longitudinal MRI study. Neurology 87:2050–2057. 10.1212/WNL.0000000000003322 PMC5109950 27733562

[12] Dincer A, Gordon BA, Hari-Raj A et al (2020) Comparing cortical signatures of atrophy between late-onset and autosomal dominant Alzheimer disease. Neuroimage Clin 28:102491. 10.1016/j.nicl.2020.102491 PMC7689410 33395982

[13] Fortea J, Sala-Llonch R, Bartrés-Faz D et al (2010) Increased cortical thickness and caudate volume precede atrophy in PSEN1 mutation carriers. J Alzheimers Dis 22:909–922. 10.3233/JAD-2010-100678 20858974

[14] Aisen PS, Jimenez-Maggiora GA, Rafii MS et al (2022) Early-stage Alzheimer disease: getting trial-ready. Nat Rev Neurol 18:389–399. 10.1038/s41582-022-00645-6 PMC8978175 35379951

[15] Jack CR, Andrews JS, Beach TG et al (2024) Revised criteria for diagnosis and staging of Alzheimer’s disease: Alzheimer’s Association Workgroup. Alzheimers Dement 20:5143–5169. 10.1002/alz.13859 PMC11350039 38934362

[16] Jessen F, Amariglio RE, Buckley RF et al (2020) The characterisation of subjective cognitive decline. Lancet Neurol 19:271–278. 10.1016/S1474-4422(19)30368-0 PMC7062546 31958406

[17] Fazekas F, Chawluk J, Alavi A et al (1987) MR signal abnormalities at 1.5 T in Alzheimer’s dementia and normal aging. AJR Am J Roentgenol 149:351–356. 10.2214/ajr.149.2.351 3496763

[18] Zilioli A, Rosenberg A, Mohanty R et al (2025) Brain MRI volumetry and atrophy rating scales as predictors of amyloid status and eligibility for anti-amyloid treatment in a real-world memory clinic setting. J Neurol 272:84. 10.1007/s00415-024-12853-9 PMC11663166 39708177

[19] Muehlboeck J-S, Westman E, Simmons A (2014) TheHiveDB image data management and analysis framework. Front Neuroinform 7:49. 10.3389/fninf.2013.00049 PMC3880907 24432000

[20] Cedres N, Ferreira D, Machado A et al (2020) Predicting Fazekas scores from automatic segmentations of white matter signal abnormalities. Aging (Albany NY) 12:894–901. 10.18632/aging.102662 PMC6977667 31927535

[21] Dakterzada F, López-Ortega R, Arias A et al (2021) Assessment of the concordance and diagnostic accuracy between Elecsys and Lumipulse fully automated platforms and Innotest. Front Aging Neurosci 13:604119. 10.3389/fnagi.2021.604119 PMC7970049 33746733

[22] Rosenberg A, Öhlund-Wistbacka U, Hall A et al (2022) β-Amyloid, Tau, neurodegeneration classification and eligibility for anti-amyloid treatment in a memory clinic population. Neurology 99:e2102–e2113. 10.1212/WNL.0000000000201043 PMC9651451 36130840

[23] Nasreddine ZS, Phillips NA, Bédirian V et al (2005) The Montreal Cognitive Assessment, MoCA: a brief screening tool for mild cognitive impairment. J Am Geriatr Soc 53:695–699. 10.1111/j.1532-5415.2005.53221.x 15817019

[24] Folstein MF, Robins LN, Helzer JE (1983) The Mini-Mental State Examination. Arch Gen Psychiatry 40:812. 10.1001/archpsyc.1983.01790060110016 6860082

[25] Bean J (2011) Rey auditory verbal learning test, Rey AVLT. In: Kreutzer JS, DeLuca J, Caplan B (eds) Encyclopedia of clinical neuropsychology. Springer, New York, NY, pp 2174–2175

[26] Caffarra P, Vezzadini G, Dieci F et al (2002) Rey-Osterrieth complex figure: normative values in an Italian population sample. Neurol Sci 22:443–447. 10.1007/s100720200003 11976975

[27] Meyers JE, Zellinger MM, Kockler T et al (2013) A validated seven-subtest short form for the WAIS-IV. Appl Neuropsychol Adult 20:249–256. 10.1080/09084282.2012.710180 23530602

[28] Kroenke K, Spitzer RL, Williams JB (2001) The PHQ-9: validity of a brief depression severity measure. J Gen Intern Med 16:606–613. 10.1046/j.1525-1497.2001.016009606.x PMC1495268 11556941

[29] Busche MA, Hyman BT (2020) Synergy between amyloid-β and tau in Alzheimer’s disease. Nat Neurosci 23:1183–1193. 10.1038/s41593-020-0687-6 PMC11831977 32778792

[30] Strikwerda-Brown C, Hobbs DA, Gonneaud J et al (2022) Association of elevated amyloid and tau positron emission tomography signal with near-term development of Alzheimer disease symptoms in older adults without cognitive impairment. JAMA Neurol 79:975–985. 10.1001/jamaneurol.2022.2379 PMC9339146 35907254

[31] Moscoso A, Heeman F, Raghavan S et al (2025) Frequency and clinical outcomes associated with tau positron emission tomography positivity. JAMA 334:229–242. 10.1001/jama.2025.7817 PMC12848857 40522652

[32] Guo T, Landau SM, Jagust WJ, Alzheimer’s Disease Neuroimaging Initiative (2020) Detecting earlier stages of amyloid deposition using PET in cognitively normal elderly adults. Neurology 94:e1512–e1524. 10.1212/WNL.0000000000009216 PMC7251521 32188766

[33] Gordon BA, Blazey TM, Su Y et al (2018) Spatial patterns of neuroimaging biomarker change in individuals from families with autosomal dominant Alzheimer’s disease: a longitudinal study. Lancet Neurol 17:241–250. 10.1016/S1474-4422(18)30028-0 PMC5816717 29397305

[34] Ossenkoppele R, Smith R, Mattsson-Carlgren N et al (2021) Accuracy of tau positron emission tomography as a prognostic marker in preclinical and prodromal Alzheimer disease: a head-to-head comparison against amyloid positron emission tomography and magnetic resonance imaging. JAMA Neurol 78:961–971. 10.1001/jamaneurol.2021.1858 PMC8240013 34180956

[35] Hartley SL, Handen B, Tudorascu D et al (2024) AT(N) biomarker profiles and Alzheimer’s disease symptomology in Down syndrome. Alzheimers Dement 20:366–375. 10.1002/alz.13446 PMC10840615 37641428

[36] Zetterberg H, Bendlin BB (2026) Biofluid biomarkers in Alzheimer’s disease and other neurodegenerative dementias. Nature 650:49–59. 10.1038/s41586-025-10018-w 41639575

[37] Tan M-S, Ji X, Li J-Q et al (2020) Longitudinal trajectories of Alzheimer’s ATN biomarkers in elderly persons without dementia. Alzheimers Res Ther 12:55. 10.1186/s13195-020-00621-6 PMC7216714 32393375

[38] Heinzinger N, Maass A, Berron D et al (2023) Exploring the ATN classification system using brain morphology. Alzheimers Res Ther 15:50. 10.1186/s13195-023-01185-x PMC10009950 36915139

[39] Ekman U, Ferreira D, Westman E (2018) The A/T/N biomarker scheme and patterns of brain atrophy assessed in mild cognitive impairment. Sci Rep 8:8431. 10.1038/s41598-018-26151-8 PMC5976713 29849029

[40] Zilioli A, Pancaldi B, Baumeister H et al (2025) Unveiling the hippocampal subfield changes across the Alzheimer’s disease continuum: a systematic review of neuroimaging studies. Brain Imaging Behav 19:253–267. 10.1007/s11682-024-00952-0 39443362

[41] Tao W, Li H, Li X et al (2021) Progressive brain degeneration from subjective cognitive decline to amnestic mild cognitive impairment: evidence from large-scale anatomical connection classification analysis. Front Aging Neurosci 13:687530. 10.3389/fnagi.2021.687530 PMC8312851 34322011

[42] Putcha D, Katsumi Y, Touroutoglou A et al (2025) Heterogeneous clinical phenotypes of sporadic early-onset Alzheimer’s disease: a neuropsychological data-driven approach. Alzheimers Res Ther 17:38. 10.1186/s13195-025-01689-8 PMC11800584 39915859

[43] Serrano-Pozo A, Das S, Hyman BT (2021) APOE and Alzheimer’s disease: advances in genetics, pathophysiology, and therapeutic approaches. Lancet Neurol 20:68–80. 10.1016/S1474-4422(20)30412-9 PMC8096522 33340485

