# Supplementary material for: Cortical thickness signature of early-onset Alzheimer’s disease across clinical stages and A/T1 biomarker profiles
Source: J Neurol. 2026 Oct 7;273(10):646. doi: 10.1007/s00415-026-14187-0 (PMC13642493; doi:10.1007/s00415-026-14187-0)
Supplement: Supplementary file 1 [file 415_2026_14187_MOESM1_ESM.docx]

**Supplementary Table S1. Availability of global and extended cognitive assessments in the study sample.**

**Panel A. Availability by biomarker profile**

| **Assessment** | **Overall (N=292)** | **Controls (N=107)** | **A+T1− (N=76)** | **A+T1+ (N=109)** |
| --- | --- | --- | --- | --- |
| MoCA | 233/292 (79.8%) | 80/107 (74.8%) | 66/76 (86.8%) | 87/109 (79.8%) |
| MMSE | 188/292 (64.4%) | 64/107 (59.8%) | 49/76 (64.5%) | 75/109 (68.8%) |
| MoCA and/or MMSE | 247/292 (84.6%) | 85/107 (79.4%) | 68/76 (89.5%) | 94/109 (86.2%) |
| Both MoCA and MMSE | 174/292 (59.6%) | 59/107 (55.1%) | 47/76 (61.8%) | 68/109 (62.4%) |
| RAVLT learning/free recall | 185/292 (63.4%) | 68/107 (63.6%) | 53/76 (69.7%) | 64/109 (58.7%) |
| RAVLT delayed recall | 183/292 (62.7%) | 67/107 (62.6%) | 54/76 (71.1%) | 62/109 (56.9%) |
| RCF copy | 158/292 (54.1%) | 62/107 (57.9%) | 43/76 (56.6%) | 53/109 (48.6%) |
| RCF delayed recall | 179/292 (61.3%) | 68/107 (63.6%) | 51/76 (67.1%) | 60/109 (55.0%) |
| WAIS-IV Coding | 180/292 (61.6%) | 68/107 (63.6%) | 51/76 (67.1%) | 61/109 (56.0%) |

**Panel B. Availability by clinical stage**

| **Assessment** | **SCD (N=148)** | **MCI (N=76)** | **Dementia (N=68)** |
| --- | --- | --- | --- |
| MoCA | 116/148 (78.4%) | 62/76 (81.6%) | 55/68 (80.9%) |
| MMSE | 88/148 (59.5%) | 53/76 (69.7%) | 47/68 (69.1%) |
| MoCA and/or MMSE | 122/148 (82.4%) | 69/76 (90.8%) | 56/68 (82.4%) |
| Both MoCA and MMSE | 82/148 (55.4%) | 46/76 (60.5%) | 46/68 (67.6%) |
| RAVLT learning/free recall | 99/148 (66.9%) | 53/76 (69.7%) | 33/68 (48.5%) |
| RAVLT delayed recall | 98/148 (66.2%) | 53/76 (69.7%) | 32/68 (47.1%) |
| RCF copy | 90/148 (60.8%) | 44/76 (57.9%) | 24/68 (35.3%) |
| RCF delayed recall | 99/148 (66.9%) | 50/76 (65.8%) | 30/68 (44.1%) |
| WAIS-IV Coding | 99/148 (66.9%) | 50/76 (65.8%) | 31/68 (45.6%) |

**Note.** Values are n/N (%), where N is the number of participants in the corresponding subgroup. Extended cognitive assessments comprised RAVLT learning/free recall, RAVLT delayed recall, RCF copy, RCF delayed recall, and WAIS-IV Coding. **Abbreviations:** MoCA, Montreal Cognitive Assessment; MMSE, Mini-Mental State Examination; RAVLT, Rey Auditory Verbal Learning Test; RCF, Rey Complex Figure Test; WAIS-IV, Wechsler Adult Intelligence Scale, Fourth Edition; SCD, subjective cognitive decline; MCI, mild cognitive impairment.
